# The acceptability of delayed consent for prehospital emergency care research in the Western Cape province of South Africa

**DOI:** 10.1371/journal.pone.0262020

**Published:** 2022-01-21

**Authors:** Willem Stassen, Sanjeev Rambharose, Lee Wallis, Keymanthri Moodley

**Affiliations:** 1 Division of Emergency Medicine, University of Cape Town, Cape Town, South Africa; 2 Department of Medicine, Centre for Medical Ethics and Law, Stellenbosch University, Stellenbosch, South Africa; University of Limpopo, SOUTH AFRICA

## Abstract

**Background:**

Informed consent is an essential prerequisite for enrolling patients into a study. Obtaining informed consent in an emergency is complex and often impossible. Delayed consent has been suggested for emergency care research. This study aims to determine the acceptability of prehospital emergency care research with delayed consent in the Western Cape community of South Africa.

**Methods:**

This study was an online survey of a stratified, representative sample of community members in the Western Cape province of South Africa. We calculated a powered sample size to be 385, and a stratified sampling method was employed. The survey was based on similar studies and piloted. Data were analysed descriptively.

**Results:**

A total of 807 surveys were returned. Most respondents felt that enrolment into prehospital research would be acceptable if it offered direct benefit to them (n = 455; 68%) or if their condition was life-threatening and the research would identify improved treatment for future patients with a similar condition (n = 474; 70%). Similar results were appreciable when asked about the participation of their family member (n = 445; 66%) or their child (n = 422; 62%) regarding direct prospects of benefit. Overwhelmingly, respondents indicated that they would prefer to be informed of their own (n = 590; 85%), their family member’s (n = 593; 84%) or their child’s (n = 587; 86%) participation in a study immediately or as soon as possible. Only 35% (n = 283) agreed to retention data of deceased patients without the next of kin’s consent.

**Conclusion:**

We report majority agreement of respondents for emergency care research with delayed consent if the interventions offered direct benefit to the research participant, if the participant’s condition was life-threatening and the work held the prospect of benefit for future patients, and if the protocol for delayed consent was approved by a human research ethics committee. These results should be explored using qualitative methods.

## Introduction

It is estimated that the development of effective emergency care systems (ECS) may prevent over half of deaths and one-third of disability from conditions that are amenable to emergency care (EC) [1]. Resolution 72.16 of the World Health Assembly in 2019, highlighted a need for the development of ECS to ensure universal health coverage [2]. Research that is contextually relevant to the setting in which the healthcare interventions are performed is essential to inform and guide the development of ECS [3,4]. While there remains limited published prehospital emergency care research overall, there are almost no studies focusing on low- and middle-income countries (LMICs) [3,4].

The quality of evidence that research yields is hierarchical [5]. Prospectively collected data and randomised controlled trials (RCTs) have long been held as the gold standard for evidence generation [5,6]. In EC, RCTs are uncommon, and data are largely collected through observational study designs [7,8]. Many challenges have been identified in the conduct of EC trials; a recent paper specifically highlights the complexity of obtaining informed consent [9].

Informed consent honours the ethical principle of maintaining a participant’s autonomy in research enrolment [10–13]. Autonomy protects the sapient condition of choice for human beings and directing their own life’s course (self-determinism), or free will [11]. By applying this principle through informed consent in research ensures that participation is voluntary and that a research participant is offered sufficient information to act by free will and make autonomous, rational decisions [11]. Autonomy and self-determinism are principles that are often protected by national legislation, for example, in the constitution of South Africa [14].

Informed consent is the process by which *“a subject* [research participant] *voluntarily confirms his or her willingness to participate in a particular trial*, *after having been informed of all aspects of the trial that are relevant to the subject’s decision to participate”* [15]. Obtaining informed consent in an emergency situation is complex and often impossible [10,16]. This may be due to the emotional state of the patient, physical symptoms, or cognitive impairment due to the patient’s injury or illness [17]. The emotional state of the proxy may also preclude obtaining informed proxy consent. Again, due to the nature of the injury or illness, there may exist a real risk to the patient’s well-being should consent procedures, including seeking consent from a proxy, delay any clinical interventions [10]. It is for this reason that “delayed” or “deferred consent” has been suggested for enrolment into emergency care research [10,16]. Research ethics guidelines from many high-income countries and LMICs make provision for this [18], including South Africa [10].

Delayed consent refers to the enrolment of a participant into clinical research before providing informed consent, because the participant lacks decision-making capacity [19]. Once the participant has recovered from their injury or illness to such an extent as to regain decision-making capacity, written informed consent is obtained to continue participation in the study and to retain data gathered for study purposes [19]. This approach has previously been used in EC trials [20].

In order to make these trials acceptable to communities, it is essential to ensure that stakeholders are engaged to discuss their views [21]. This may, at least in part, honour respect for the autonomy of a community.

Previous research interrogated the acceptability of delayed consent trials in EC research and found that only half of those surveyed thought research without informed consent was acceptable [22]. In a larger study, EC research with delayed consent was less supported, with 35% of respondents rejecting the concept [23]. Interestingly, the application of the concept, however, was acceptable to half of the respondents surveyed should the respondent be involved in such a study themselves [23]. When the notion was explained within the context of an actual study, acceptability increased to 82% [23]. Clear cultural distinctions have been demonstrated related to the acceptability of the delayed consent [24], and acceptability was influenced by the type of study and the perceived level of risk [25].

The results of these studies support the notion that the acceptability of delayed consent trials is relative to the specific socio-cultural contexts and the types of interventions being studied. Within the diverse South African milieu, with eleven official languages, differing levels of education and religious heterogeneity, obtaining informed consent for research is complicated, at best. For these reasons, findings are unlikely to be transferable to the South African and Western Cape setting, as ethical convictions may be related to a specific culture or individual and are therefore not generalisable to people who do not share the same cultures or belief systems [11]. Apart from three publications related to posthumous intensive care research with a waiver and delayed proxy consent in the context of public health emergencies [26–28], a literature search did not yield any specific studies related to delayed consent studies in EC in the South African context. This study, therefore, aims to fill this knowledge gap and to determine whether members of the Western Cape community of South Africa find prehospital emergency care research with delayed consent acceptable.

## Methods

We employed a quantitative, cross-sectional online survey design of a stratified, representative sample of community members in the Western Cape. This survey forms the quantitative phase of an exploratory sequential mixed-methods study.

### Setting

The Western Cape has a population of 6.5 million people [29]. As culture, language and religion may all affect ethical convictions [30] this information was essential to guide sampling to ensure representation of the Western Cape population.

When we consider the demographic profile of the province according to religion and language, the following is apparent:

- Religion: Christian (89%), Muslim (7%), Undefined (1.6%), African religions (1%), Jewish (0.3%), Atheist/Agnostic (0.3%) [31]
- Language: Afrikaans (50%), English (20%), isiXhosa (25%), Other (5%) [32]
- Medical insurance: Yes (26%), No (74%)

### Sample and sampling

Owing to the requirements for physical distancing and lack of access to public spaces during the COVID-19 pandemic, we conducted the survey online. The survey was hosted on the AfricanPulse Survey (Nudge Insights, Rivonia, South Africa) platform and distributed to their membership via email and SMS. AfricanPulse is a free online community where anyone can join on a volunteer basis. The community is specifically designed so that members can participate in discussions on various topics, brands, and products. Invitations were also distributed via social media. All respondents consented to participate in the survey by reviewing a digital informed consent form and indicating their consent by means of a mandatory field tick box. The details of the investigators were provided at the outset should respondents have any questions or concerns.

Assuming a target population of 5.6 million people, we accepted a margin of error of 5%, a response distribution of 50% and a confidence interval of 95%, which yielded a minimum sample size of n = 385. This sample was further stratified according to the Western Cape demographic profile, based on religion (Christian, n = 343; Muslim, n = 27; Undefined or other, n = 11; African religions, n = 4), language (Afrikaans, n = 193; English, n = 77; isiXhosa, n = 96; Other, n = 19) and medical insurance status (Medical insurance: Yes, n = 100; No, n = 285).

A stratified sampling technique was employed to ensure that the sampled population is representative of the demographic profile of the province. This was done by sending targeted invitations to AfricanPulse members and using geolocation on social media platforms. Finally, a series of screening questions before the survey was undertaken ensured that respondents were residents of the Western Cape.

### Survey instrument

The survey instrument was based on similar studies [23,24,33] conducted internationally through a validated survey method. Face validity of the survey was established by review from experts in South African EC and survey design. The survey was also piloted by a community representative who has functioned as a community liaison officer in the province.

The survey was divided into two sections. In the first section, the survey ascertained the demographic profile of the participant, as these may affect culture and thus, ethical convictions [30]. The second section of the survey aimed to determine the acceptability of emergency care research with delayed consent in a variety of different situations expressed as statements. Situations related to the likelihood of benefit as well as acting as different consenting agents (self, proxy, parent). Agreement with these statements was assessed using a five-point Likert-type scale (strongly agree to strongly disagree). The survey was available in English, Afrikaans, and isiXhosa.

### Data analysis

Data were subjected to descriptive analysis and expressed as proportions of agreement (strongly agree, agree) or disagreement (strongly disagree, disagree) with statements. Neutral was not allocated to either of these categories. Respondents were not obligated to answer all statements, and proportions are therefore expressed using the number of valid responses as the denominator.

Ethical approval was obtained from the Human Research Ethics Committee of the University of Stellenbosch (ref nr: N19/08/114).

## Results

A total of 807 respondents completed the online survey, 4129 invitations were sent to AfricanPulse members. While most respondents chose to answer the survey in English (n = 707; 88%), 62% (n = 498) of respondents reported their mother tongue to be English, 25% (n = 198) to be Afrikaans and 12% (n = 98) to be isiXhosa, respectively. Table 1 below outlines the demographic profile of the respondents, cross-tabulated with whether the participant had been transported by ambulance before. Tables 2–4 show the results of the survey items.

**Table 1:** Demographic details of respondents.

	TRANSPORTED IN AMBULANCE		
	Yes	No	Total
*Total*	*344 (43%)*	*461 (57%)*	*805*
**Self-reported gender**			
Male	143 (42%)	165 (36%)	308 (38%)
Female	199 (58%)	292 (63%)	493 (61%)
Prefer not to answer	2 (1%)	4 (1%)	6 (1%)
**Marital status**			
Married / Living with a partner	152 (44%)	214 (46%)	366 (45%)
Separated / Divorced	49 (14%)	39 (8%)	88 (11%)
Widowed	19 (6%)	19 (4%)	39 (5%)
Single	121 (35%)	188 (41%)	309 (38%)
Did not answer	3 (1%)	1 (0%)	4 (1%)
**Religion**			
Christian	279 (81%)	349 (76%)	630 (78%)
Muslim	27 (8%)	60 (13%)	87 (11%)
African Religion	8 (2%)	14 (3%)	22 (3%)
Jewish	2 (1%)	3 (1%)	5 (1%)
Atheist / Agnostic	10 (3%)	11 (2%)	21 (3%)
Other	11 (3%)	14 (3%)	25 (3%)
Rather not say	7 (2%)	10 (2%)	17 (2%)
**Highest level of education**			
Completed primary school	9 (3%)	8 (2%)	17 (2%)
Completed Grade 9 / Standard 7	58 (17%)	67 (15%)	125 (15%)
Completed high school	147 (43%)	173 (38%)	320 (40%)
Higher education / College	39 (11%)	80 (17%)	120 (15%)
National Certificate	28 (8%)	29 (6%)	57 (7%)
National Diploma	38 (11%)	39 (8%)	77 (10%)
Bachelor’s Degree	21 (6%)	50 (11%)	71 (9%)
Master’s Degree	2 (1%)	12 (3%)	14 (2%)
Doctorate Degree	2 (1%)	0 (0%)	2 (0%)
Did not answer	0 (0%)	3 (1%)	3 (0%)

**Table 2:** Views on emergency care research.

	Strongly Agree	Agree	Neutral	Disagree	Strongly Disagree
Healthcare workers who conduct research can be trusted to act in the best interests of the participant.	186 (29%)	245 (39%)	164 (26%)	29 (5%)	9 (1%)
If I participate in medical research, there are enough protections to ensure that I will be treated safely and with minimal risk to my well-being.	218 (34%)	218 (34%)	155 (24%)	36 (6%)	9 (1%)
It is important to conduct ongoing research in emergency medicine.	345 (54%)	236 (37%)	45 (7%)	9 (1%)	2 (0%)
It is important to conduct ongoing research in prehospital emergency care.	275 (44%)	269 (43%)	72 (11%)	10 (2%)	4 (1%)
The benefits of participating in prehospital emergency care research outweigh the risks.	123 (20%)	217 (35%)	222 (36%)	45 (7%)	17 (3%)
All research participants should be informed about the study prior to being entered into a study.	389 (61%)	209 (33%)	34 (5%)	2 (0%)	1 (0%)
All research participants should have the option to decline research participation.	301 (48%)	241 (38%)	55 (9%)	19 (3%)	10 (2%)
There are situations in which it is so important to learn about a new treatment that it is okay to enrol patients in a study without their permission.	91 (14%)	112 (18%)	105 (17%)	164 (26%)	156 (25%)
I would support emergency medicine research which has been approved by an ethics committee but involves starting treatment before consent can be obtained.	140 (22%)	226 (36%)	128 (20%)	99 (16%)	38 (6%)
I would feel that I was helping future generations.	281 (44%)	281 (44%)	64 (10%)	3 (0%)	5 (1%)
I would feel that taking part could lead to better medical treatments.	272 (43%)	292 (46%)	68 (11%)	5 (1%)	1 (0%)
I would feel that taking part would help the doctors, whom I get my medical care from, take better care of other patients.	299 (47%)	274 (43%)	52 (8%)	9 (1%)	2 (0%)
I would feel that taking part could help my family.	221 (34%)	301 (47%)	103 (16%)	16 (2%)	2 (0%)
I would feel that taking part could help me personally.	217 (34%)	269 (42%)	131 (21%)	15 (2%)	3 (0%)
I would worry about my privacy.	178 (28%)	229 (36%)	134 (21%)	75 (12%)	13 (2%)
I would worry about my medical record being shared.	165 (26%)	216 (34%)	147 (23%)	83 (13%)	22 (3%)
I would worry about how researchers would use my health information.	170 (27%)	218 (34%)	155 (24%)	76 (12%)	21 (3%)
I would worry that some research would be done that I did not want to take part in.	159 (25%)	251 (39%)	149 (23%)	63 (10%)	16 (3%)
I would worry that someone might make money using my health information.	185 (30%)	183 (29%)	161 (26%)	76 (12%)	22 (4%)
I would want to know what kind of knowledge would result from my participation	330 (52%)	259 (40%)	46 (7%)	2 (0%)	3 (0%)
I would want to know who guarantees that my health information is protected.	354 (56%)	227(36%)	43 (7%)	8 (1%)	4 (1%)

**Table 3:** Views on delayed consent for prehospital emergency care research under various conditions.

	Strongly Agree	Agree	Neutral	Disagree	Strongly Disagree
**In general, it would be okay to enrol patients in prehospital research without their permission if…**					
The patient is unable to provide permission, and no surrogate is available to speak for the patient.	114 (17%)	227 (34%)	168 (25%)	104 (15%)	62 (9%)
The research study offers direct benefit to the patient.	182 (27%)	287 (42%)	116 (17%)	50 (7%)	42 (6%)
The research study offers no direct benefit to the patient but could benefit others in the future.	100 (15%)	206 (30%)	172 (25%)	139 (21%)	73 (11%)
The patient’s condition is life-threatening and the research will help identify a better way of treating future patients with a similar condition.	218 (32%)	283 (41%)	110 (16%)	46 (7%)	31 (5%)
**I would be willing to participate in a prehospital research study without giving my consent if…**					
I am unable to provide permission and no surrogate is available to speak for me.	121 (18%)	225 (33%)	146 (22%)	120 (18%)	63 (9%)
The research study offers direct benefit to the patient.	180 (27%)	275 (41%)	126 (19%)	52 (8%)	41 (6%)
The research study offers no direct benefit to me but could benefit others in the future.	119 (18%)	210 (31%)	165 (24%)	111 (16%)	69 (10%)
My condition is life-threatening, and the research will help identify a better way of treating future patients with a similar condition.	203 (30%)	271 (40%)	122 (18%)	55 (8%)	35 (5%)
**It would be okay to enrol my family member in prehospital research without their consent if…**					
My family member is unable to give their permission and no surrogate is available to speak for them.	103 (15%)	202 (30%)	178 (26%)	135 (20%)	63 (9%)
The research study offers direct benefit to my family member.	173 (26%)	272 (40%)	118 (18%)	62 (9%)	48 (7%)
The research study offers no direct benefit to my family member but could benefit others in the future.	90 (13%)	197 (28%)	193 (28%)	139 (20%)	74 (11%)
**It would be okay to enrol my child in prehospital research without their consent if…**					
I am unable to give my permission and no surrogate is available to speak for my child or me.	82 (12%)	172 (25%)	153 (22%)	131 (19%)	101 (15%)
The research study offers direct benefit to my child.	159 (23%)	263 (39%)	130 (19%)	46 (7%)	45 (7%)
The research study offers no direct benefit to my child but could benefit others in the future.	93 (13%)	216 (30%)	166 (23%)	96 (14%)	85 (12%)

**Table 4:** Timing of seeking delayed consent.

	Immediately (as soon as possible)	After some time has passed	After recovery	Never
When would you like to be informed about your own enrolment in a study?	590 (85%)	30 (4%)	30 (4%)	14 (2%)
When would you like to be informed about your family member’s enrolment in a study?	593 (84%)	28 (4%)	32 (5%)	15 (2%)
When would you like to be informed about your child’s enrolment in a study?	587 (86%)	22 (3%)	22 (3%)	19 (3%)

Just under half (n = 344; 43%) of the respondents had been transported by an ambulance before (Table 1). The majority of respondents were female (n = 493; 61%), Christian (n = 630; 78%) married or living with a partner (n = 366; 45%) and had completed high school (n = 320; 40%). More than half (n = 439; 54%) of the respondents were unemployed, while 69% (n = 556) did not have medical insurance. Most (n = 488; 60%) respondents were between the ages of 31 and 60 years old (not shown in table).

Table 2 outlines the views of respondents on EC research. Almost all respondents agreed that emergency medicine (n = 581; 91%) and prehospital EC (n = 544, 87%) research was important. While most respondents agreed that everyone should have the option to decline research participation (n = 542, 86%), the majority stated that they would support emergency care research without consent should it be approved by a human research ethics committee (n = 366; 58%). These views seem to be motivated by the perception that their participation would help future generations (n = 562; 88%), future patients (n = 573; 90%), their families (n = 522; 81%) or carried the prospect of direct benefit to the participant themselves (n = 486; 76%). However, respondents expressed worry about the privacy of their health information and sought clarity on exactly what knowledge their participation would produce.

Table 3 outlines the views of respondents on prehospital EC research with delayed consent under various conditions. The majority of respondents felt that their own enrolment into prehospital EC research would be acceptable if the research offered direct benefit to them (n = 455; 68%) or if their condition was life-threatening and the research would help identify better means of treating future patients with a similar condition (n = 474; 70%). Similar results were also appreciable when respondents were asked about the participation of their family member (n = 445; 66%) or their child (n = 422; 62%) regarding direct prospects of benefit.

Table 4 shows the timing within which delayed consent should be sought. Overwhelmingly, respondents indicated that they would prefer to be informed of their own (n = 590; 85%), their family member’s (n = 593; 84%), or their child’s (n = 587; 86%) participation in a research study immediately or as soon as possible. Lastly, only 35% (n = 283) agreed that it would be acceptable to retain data of patients who have died without the next of kin’s consent.

## Discussion

This study aimed to determine whether members of the Western Cape community of South Africa find prehospital EC research with delayed consent acceptable. Designed to be statistically representative of the Western Cape demographic, we found high levels of support for delayed consent in prehospital EC research. Delayed consent was generally acceptable to respondents if the interventions offered direct benefit to the research participant, if the participant’s condition was life-threatening and the work held the prospect of benefit for future patients, and if the protocol for delayed consent was approved by a human research ethics committee. Our findings on the acceptability of delayed consent for EC research are comparable to research done in other settings, such as Australia (62%) [33], Canada (76%) [34], and the United States (70%) [35]. A recent systematic review also showed comparable results in pediatric EC research (68%) [36].

In keeping with previous studies from other settings [33,37,38], the support for delayed consent seemed to improve if there are prospects of direct benefit to the research participant themselves. This is an important consideration for human research ethics committees when deliberating whether to provide approval for studies that are proposing delayed consent. The challenge, however, is to determine which benefits can be considered as “direct” and what the magnitude of such benefits should be in order to justify the research. Additionally, in instances of scientific equipoise, it might not always be apparent what the likelihood of potential benefit could be. The failure to adequately explore this concept may either cause unnecessary barriers to important EC research or otherwise fail to balance the risk to potential respondents. We suggest that this be considered on a per-study basis and in consultation with the community.

Notwithstanding direct benefit, respondents seemed to also be motivated by altruism and deemed delayed consent for EC research acceptable should their condition be life-threatening and if the research may benefit future patients with a similar condition. This was identified in other studies, too [38]. The idea is certainly in line with the Declaration of Helsinki, which makes provision for the enrolment of study respondents with delayed consent even if no direct benefit is likely, but the research “*is intended to promote the health of the group represented by the potential subject*,*”* and “*this group should stand to benefit from the knowledge*, *practices or interventions that result from the research*.” [12] However, the responsibility for the protection of such respondents is then placed squarely on the ethics committee who are to provide approval [10].

Respondents reported that consent is sought immediately (or as soon as possible) after their own or next of kin’s enrolment in a study. This is an important consideration for the planning of future studies as it might have an impact on the study staff resources–it would not be acceptable for consent procedures to distract the clinical study staff from providing EC and thus, a separate staff member should be delegated this task. Additionally, it may be argued that a patient or proxy who finds themselves faced with an emergency may not truly be in a state of mind to adequately provide true *informed* consent [10]. It has also been shown that proxies make decisions based on what they hope might happen rather than an assessment of the risks and benefits of the research [39]. This may be cause for concern related to a therapeutic misconception, which has previously been highlighted in EC studies [40]. It is thus important that this be reviewed in community engagement and future qualitative work to better determine the exact timing of seeking consent.

Respondents generally did not consider it acceptable to retain post-mortem data without the consent of the next of kin. Excluding respondents who do not give consent because of death might create considerable bias in results by falsely elevating (if most excluded deaths occur in the intervention arm) or lowering (if most excluded deaths occur in the control arm) the efficacy of the intervention. This provides tremendous risk to the integrity of EC clinical trials. It has been suggested that this view may also be held because of altruism or the knowing that their family’s data might be able to help others in the future [40]. Previous studies have also reported mixed results regarding the wishes of bereaved families for disclosure of their family member’s involvement in research [40]. Again, we suggest that the terms of this to be discussed during community engagement and the means by which consent may be obtained should be explored. Another approach may be to obtain advanced directives for emergency care research and retention of data post-mortem during community engagement [28].

Lastly, this study provided generic questions related to delayed consent and that were not linked to any type of intervention or study. The perceived risk and benefit of a particular intervention have been shown to influence views on delayed consent more so than the principle of delayed consent [41]. It can be argued that should the research interventions be particularly relevant or important to the population being surveyed, that even higher levels of support for delayed consent in EC research could be garnered. In so doing, it also highlights the importance of community benefit as outlined in the Declaration of Helsinki [12].

### Limitations

This study is limited by its sampling strategy, specifically, that only respondents who were registered on the online survey platform and had internet access were eligible for participation. Despite this, the socio-demographic, cultural, and linguistic profiles of the provinces were well represented. This study is further limited by its quantitative design and cannot fully explain results, yet only reports on the preliminary results of a mixed-methods study.

## Conclusion

We generally report high levels of agreement from the majority of respondents for EC research with delayed consent if the interventions offered direct benefit to the research participant, if the participant’s condition was life-threatening and the work held the prospect of benefit for future patients, and if the protocol for delayed consent was approved by a human research ethics committee. Future qualitative work should explore what direct benefits should comprise of the timing of consent and discuss the conditions of seeking consent after a research participant has died.

## Supporting information

S1 File Online survey instrument administered. (PDF)
