# Supplementary material for: The acceptability of delayed consent for prehospital emergency care research in the Western Cape province of South Africa
Source: PLoS One. 2022 Jan 21;17(1):e0262020. doi: 10.1371/journal.pone.0262020 (PMC8782530; doi:10.1371/journal.pone.0262020)
Supplement: S1 File — (PDF) [file pone.0262020.s001.pdf]

PERCEPTIONS ON THE ACCEPTABILITY OF EMERGENCY CARE RESEARCH WITH DELAYED CONSENT IN THE WESTERN CAPE PROVINCE OF SOUTH AFRICA

Your responses will remain anonymous and will never be linked to you. Please complete the survey to the best of your ability. If there are items you do not feel comfortable answering, please skip them. Your participation is entirely voluntary. Thank you for your cooperation.

Please mark the appropriate response:

1. What is your AGE? _____
2. What is your GENDER?

   ☐ Male ☐ Female ☐ Other ☐ Prefer not to answer

3. What is your MARITAL STATUS?

   ☐ Married ☐ Separated/Divorced ☐ Widowed ☐ Single

4. What is your COUNTRY OF BIRTH?

   ☐ South Africa ☐ Other

5. What PROVINCE do you currently live in?

   ☐ Western Cape Province ☐ Other

6. What SUBURB do you currently live in? ________________________________

7. What is your RELIGION?

   ☐ Christian ☐ Muslim ☐ African Religion ☐ Jewish ☐ Atheist/Agnostic
   ☐ Other ☐Rather not say

8. What is your PRIMARY LANGUAGE?

   ☐ Afrikaans ☐ English ☐ isiXhosa ☐ Other

9. What is your highest level of EDUCATION?

   ☐ Completed primary school ☐ Completed grade 9/Standard 7
   ☐ Completed matric ☐ Higher education/college
   ☐ National Certificate ☐ National Diploma
   ☐ Bachelors Degree ☐ Masters Degree
   ☐ Doctorate Degree

10. Are you current EMPLOYED?

☐ Yes ☐ No

11. What is your current monthly HOUSEHOLD INCOME?
R______________________________

☐ Rather not say

12. Do you currently have MEDICAL INSURANCE?

☐ Yes ☐ No

13. Have you ever participated in medical research?

☐ Yes ☐ No ☐ I refused to participate

If you answered "I refused to participate," please explain:

____
____
____

14. Have you ever given permission for someone else (minor or incapacitated family) to participate in research?

☐ Yes ☐ No ☐ I refused to give permission

If you answered "I refused to give permission," please explain:

____
____
____

15. Have you ever been transported by an ambulance service?

☐ Yes ☐ No

16. Has anyone you know ever been transported by an ambulance service?

☐ Yes ☐ No

Please indicate your level of agreement or disagreement with the following statements:

**Please indicate your level of agreement or disagreement with the following statements:**	Strongly Disagree	Disagree	Neutral	Agree	Strongly Agree
Healthcare workers who conduct research can be trusted to act in the best interests of the participant.	☐	☐	☐	☐	☐
If I participate in medical research, there are enough protections to ensure that I will be treated safely and with minimal risk to my well-being.	☐	☐	☐	☐	☐
It is important to conduct ongoing research in emergency medicine.	☐	☐	☐	☐	☐
It is important to conduct ongoing research in pre-hospital emergency care.	☐	☐	☐	☐	☐
The benefits of participating in pre-hospital emergency care research outweigh the risks.	☐	☐	☐	☐	☐
All research participants should be informed about the study prior to being entered into a study.	☐	☐	☐	☐	☐
All research participants should have the option to decline research participation.	☐	☐	☐	☐	☐
There are situations in which it is so important to learn about a new treatment that it is okay to enrol patients in a study without their permission.	☐	☐	☐	☐	☐
I would support emergency medicine research which has been approved by an ethics committee but involves starting treatment before consent can be obtained.	☐	☐	☐	☐	☐

Please indicate your level of agreement or disagreement with the following statements:

**If I were to participate in a Research Study…**	Strongly Disagree	Disagree	Neutral	Agree	Strongly Agree
…I would feel that I was helping future generations.	☐	☐	☐	☐	☐
…I would feel that taking part could lead to better medical treatments.	☐	☐	☐	☐	☐
…I would feel that taking part would help doctors where I get my medical care take better care of other patients.	☐	☐	☐	☐	☐
…I would feel that taking part could help my family.	☐	☐	☐	☐	☐
…I would feel that taking part could help me personally.	☐	☐	☐	☐	☐
…I would worry about my privacy.	☐	☐	☐	☐	☐
…I would worry about my medical record being shared.	☐	☐	☐	☐	☐
…I would worry about how researchers would use my health information.	☐	☐	☐	☐	☐
…I would worry that some research would be done that I did not want to take part in.	☐	☐	☐	☐	☐
…I would worry that someone might make money using my health information.	☐	☐	☐	☐	☐
…I would want to know what kind of knowledge would result from my participation	☐	☐	☐	☐	☐
…I would want to know who guarantees that my health information is protected.	☐	☐	☐	☐	☐

Please indicate your level of agreement or disagreement with the following statements:

**In general, it would be okay to enrol patients in pre-hospital research without their permission if…**	Strongly Disagree	Disagree	Neutral	Agree	Strongly Agree
…the patient is unable to provide permission and no surrogate is available to speak for the patient.	☐	☐	☐	☐	☐
… the research study offers direct benefit to the patient.	☐	☐	☐	☐	☐
… the research study offers no direct benefit to the patient but could benefit others in the future.	☐	☐	☐	☐	☐
… the patient's condition is life threatening and the research will help identify a better way of treating future patients with a similar condition.	☐	☐	☐	☐	☐
**I would be willing to participate in a pre-hospital research study without giving my consent if…**	Strongly Disagree	Disagree	Neutral	Agree	Strongly Agree
… I am unable to provide permission and no surrogate is available to speak for me.	☐	☐	☐	☐	☐
… the research study offers direct benefit to the patient.	☐	☐	☐	☐	☐
… the research study offers no direct benefit to me but could benefit others in the future.	☐	☐	☐	☐	☐
… my condition is life threatening and the research will help identify a better way of treating future patients with a similar condition.	☐	☐	☐	☐	☐
**It would be okay to enrol my family member in pre-hospital research without their consent if…**	Strongly Disagree	Disagree	Neutral	Agree	Strongly Agree
… my family member is unable to give their permission and no surrogate is available to speak for them.	☐	☐	☐	☐	☐
… the research study offers direct benefit to my family member.	☐	☐	☐	☐	☐
… the research study offers no direct benefit to my family member but could benefit others in the future.	☐	☐	☐	☐	☐

… my family member's condition is life threatening and the research will help identify a better way of treating future patients.	☐	☐	☐	☐	☐
**It would be okay to enrol my child in pre-hospital research without their consent if…**	Strongly Disagree	Disagree	Neutral	Agree	Strongly Agree
… I am unable to give my permission and no surrogate is available to speak for me or my child.	☐	☐	☐	☐	☐
… the research study offers direct benefit to my child.	☐	☐	☐	☐	☐
… the research study offers no direct benefit to my child but could benefit my child in the future.	☐	☐	☐	☐	☐
	Strongly Disagree	Disagree	Neutral	Agree	Strongly Agree
If a patient who was part of a research study dies and information about their treatment is used in the study, I think it would be acceptable to use the data without the families' consent	☐	☐	☐	☐	☐
Regarding timing of delayed consent…	Immediately (as soon as possible)	After some time has passed	After recovery	Never	Unsure/ Not answered
… when would you like to be informed about your own enrolment in a study?	☐	☐	☐	☐	☐
… when would you like to be informed about your family member's enrolment in a study?	☐	☐	☐	☐	☐
… when would you like to be informed about your child's enrolment in a study?	☐	☐	☐	☐	☐
… when would you like to be informed about your family member's enrolment in a study, if they passed away?	☐	☐	N/A	☐	☐
